# Lost in machine translation: The promises and pitfalls of machine translation for multilingual group work in global health education

**DOI:** 10.1007/s44217-022-00004-z

**Published:** 2022-05-16

**Authors:** David C. Hill, Christy Gombay, Otto Sanchez, Bethel Woappi, Andrea S. Romero Vélez, Stuart Davidson, Emma Z. L. Richardson

**Affiliations:** 1 Global Health Office, McMaster University, Hamilton, Canada; 2 Dalla Lana School of Public Health, University of Toronto, Toronto, Canada; 3 Faculty of Health Sciences, Ontario Tech University, Oshawa, Canada; 4 Universidad del Rosario, Bogotá, Colombia; 5 Department of Health Research Methods, Evidence and Impact, McMaster University, Hamilton, Canada

## Abstract

The rapid adoption of online technologies to deliver postsecondary education amid the COVID-19 pandemic has highlighted the potential for online learning, as well as important equity gaps to be addressed. For over ten years, McMaster University has delivered graduate global health education through a blended-learning approach. In partnership with universities in the Netherlands, India, Thailand, Norway, Colombia, and Sudan, experts from across the Consortium deliver lectures online to students around the world. In 2020, two courses were piloted with small groups of students from Canada and Colombia using machine translation supported by bilingual tutors. Students met weekly via video conferencing software, speaking in English and Spanish and relying on machine translation software to transcribe and translate for group members. Qualitative semi-structured interviews were conducted with students, tutors, and instructors to explore how artificial intelligence can be harnessed to integrate multilingual group work into course offerings, challenging the dominant use of English as the principal language of instruction in global health education. Findings highlight the potential for machine translation to bridge language divides, while also underscoring several key limitations of currently available technology. Further research is needed to investigate the potential for machine translation in facilitating multilingual online education as a pathway to more equitable and inclusive online learning environments.

## Introduction

The COVID-19 pandemic forced postsecondary institutions around the world to transition rapidly to online delivery of graduate education [1–4]. For many, the transition was uneven and jolting, with issues ranging from technical challenges, such as unreliable internet connectivity, to cumbersome digital platforms, and lack of familiarity with digital technologies [5–7]. Extensive literature also documents the ‘digital divide’, or the differential access to, and ability to use, modern information and communication technologies [8, 9], which reinforces prevailing class and social inequities both within and across countries [10–12]. The transition to online education amid COVID-19 may exacerbate these inequities, so educators should consider these barriers when planning and implementing online teaching. There is value in looking to the experiences of educators delivering global and public health education online prior to COVID-19 to forge a path forward.

In 2010, McMaster University (Canada) and Maastricht University (The Netherlands) formed a higher education Consortium that researches and teaches globalization and health [13]. Consortium members include Manipal Academy of Higher Education (India), Thammasat University (Thailand), the University of South-Eastern Norway (Norway), Universidad del Rosario (Colombia) and Ahfad University for Women (Sudan), capitalizing on the expertise at each higher education institution to offer a unique model for graduate education. Each institution offers its own graduate program in global or public health, with two core courses shared across the Consortium, Foundations of Global Health I and II. In these two courses, global health experts from across the Consortium deliver lectures, which are streamed via video conferencing software for students around the world to watch synchronously and are recorded for students to stream asynchronously at their convenience. Group work is also central to these courses; McMaster students are assigned to groups with students from Maastricht University or Universidad del Rosario. Groups meet online weekly with a tutor to review course readings, discuss lecture material, and complete group assignments.

## Machine translation as a teaching tool for multilingual group work

While addressing issues of equity in bridging the digital divide across age, gender, race, and class have proved important, our research brings into focus a more subtle equity gap to be addressed in global health education: the de facto use of English as the primary language of instruction, and the additional intellectual labour this imposes on non-Anglophone learners. For example, non-Anglophone learners described additional time spent re-reading course materials, seeking literature in their native languages to support their understanding, and working with online translation tools to complete assignments in English. Non-Anglophone learners also described reviewing lecture recordings multiple times to understand course materials, as well as a reluctance to lead, or even participate in, small group settings due to language barriers. In response, in 2020 the Consortium piloted the use of machine translation as a tool to facilitate multilingual group work with small groups of students from Canada and Colombia, supported by bilingual tutors. Colombian students were encouraged to participate in group meetings fully in Spanish, utilizing *Microsoft Translator: Conversations* [14] to translate and transcribe speech for English-speaking group members. Tutors attended group meetings to support with translation, clarify misunderstandings, and explain course concepts. While previous research has explored the use of machine translation to support language learning [15, 16], and teaching in a primary-school setting [17], limited research explores the implementation of machine translation in post-secondary education.

Implementing this transnational, multilingual, blended-learning initiative has not been without challenges. Coordinating group meetings involves navigating five time zones, and for many students is the first time working virtually in international groups. Tutors are from across the Consortium, so regular communication between course coordinators and tutors was crucial to ensure consistency across groups. Moreover, licencing for required learning management software was negotiated at each institution to ensure all students had access to lectures and tutorials, which necessitated Maastricht University undertaking additional administrative labour. There are also ongoing dilemmas surrounding how to update curricula in a course shared by six universities, three of which are in the global South.

Montenegro et al. [18] argue that although in recent years there have been calls to decolonize global health interventions and acknowledge colonial power relations in global health academia [19], less focus has been paid to decolonizing global health education. This endeavour requires an intentional challenging of the effects of colonialism which has reinforced inequitable power relations in global health education [20]. We acknowledge the tension in writing about decolonizing global health education from a global North university, but this brief communication introduces key findings from a qualitative study exploring the experiences of students, tutors, and instructors utilizing machine translation in global health education. We are now at a pinnacle moment, both with the potential for artificial intelligence technology to facilitate multilingual group work, and with the rapid global transition to online learning, to assess the potential for machine translation to circumvent inequities produced by using English as the principal language of instruction in global health education.

Recent innovations in artificial intelligence (AI), and specifically machine translation, show promise for the creation of more inclusive and equitable online learning spaces in global health by allowing learners to speak their native language. At its core, AI refers to the ability of machines to carry out complex tasks typically requiring human intelligence [21]. Supported by AI, machine translation requires only a computer, smartphone, or device connected to the internet and allows for intelligent and nuanced translations. While little is known about the mechanics and outcomes of courses using machine translation, Australian and Indonesian medical students engaging in online communication tools and platforms identified real-time machine translation as a tool that facilitated fluid conversations between non-English-speaking and English-speaking groups [22]. This research thus builds on the existing literature by exploring the application of machine translation software to a higher education setting and contributes to critical reflections on the promises and pitfalls of automatic transcription and translation in global health education.

## Methodology and methods

Research design followed a realist evaluation [23] to evaluate what works, for whom, under what circumstances, and why. Qualitative, semi-structured interviews were used to assess: (1) how machine translation can be harnessed to improve online, international global health education, and (2) to explore the experiences of students and tutors utilizing machine translation software to conduct multilingual group work. In semi-structured interviews, researchers have sufficient flexibility to “follow up on particularly interesting avenues that emerge” ([24], p. 10) to gain detailed insights into respondents’ accounts of a topic. Global Health students, tutors, and program directors who were involved in the multilingual pilot project in 2020–2021 were invited to participate. Semi-structured interviews were conducted in either Spanish or English via video conferencing software with 11 participants, including three program coordinators from McMaster University, Maastricht University, and Universidad del Rosario, one tutor from McMaster University, two students from Universidad del Rosario and five students from McMaster University. Given the targeted research questions and depth of information held by participants, this sample size was anticipated to hold sufficient “information power” [25] to address research aims. Interviews were conducted via video conferencing software (Zoom), audio recorded, and transcribed verbatim. Thematic analysis [26] was utilized to identify, analyze, and report themes identified in the data. Initial sets of themes were reviewed independently by two research team members who met to discuss and develop a set of codes and their accompanying definitions, which was used to produce a structured code book. Codes were organized and applied to transcripts by two research team member using qualitative analysis software Dedoose [27], and any discrepancies were reviewed by a third member.

## Results

### The Promises and Pitfalls of Machine Translation

Preliminary findings illustrate the potential to capitalize on the strengths of machine translation to reduce inequities produced by teaching global health predominantly in English. Partnering students from McMaster University (Canada) and Universidad del Rosario (Colombia) together in virtual learning pods with tutors who are fluent in Spanish, groups communicated using *Microsoft Translator: Conversations*. McMaster students, often with limited skills in written and oral Spanish, communicated with Spanish-speaking group members using machine translation, and with the assistance of the tutor. Students were resourceful and adaptable in navigating multiple communication technologies, relying on *WhatsApp* for group communication, *Google Docs* to collaborate on assignments, *Zoom* or *Google Meets* for group meetings, *Google Translate* for text translation, and *Microsoft Translator: Conversations* for live translation during group meetings. Students emphasized that the multilingual component of group work contributed to “improved communication skills and ability to work in groups” and described multilingual group work as “richer” than previous experiences with group work. Students also described learning about other cultures as an important outcome of multilingual group work, which was noted during interviews as “essential” for work in global and public health. Canadian students emphasized gaining greater understandings of health challenges in Colombia, while Colombian students noted the ability to work with global North institutions as crucial for future work with multilateral funding agencies.

Significant limitations with currently available machine translation technologies were noted across the interviews, with *Microsoft Translator: Conversations* being described as “ineffective”, “time-consuming”, and “inaccurate”. For example, a McMaster student described the technology as “[not] effective at all. The translation wasn’t very good and it was very slow”. Similarly, a Rosario student explained how the group “lo [intentaron] usar pero fue muy complejo” [tried to use the translator but it was very complicated]. In line with the literature on the digital divide [8], some students noted their age or lack of familiarity with technologies as key barriers in adopting machine translation software. One student noted that “maybe the other younger students in the class found it easier. But for me, it was quite challenging to even start to know how to use some of the software”. Given these challenges, some groups abandoned translation technologies and relied solely on bilingual tutors and group members to translate. Students also underscored the challenges in translating cultural contexts that do not necessarily have a linguistic equivalent in English, resulting in local meanings being lost in machine translation. For example, Colombian students described difficulties in translating concepts from Indigenous traditional medicines, and often relied on terminology from Western medicine when translating to English:

también siento que, desde la epistemología, porque el tema que estábamos tratando era de medicinas tradicionales...entonces siento que había unos temas que ellas comprendían desde la lógica del norte, de la ciencia, que no tienen la misma explicación desde una lógica indígena, o una lógica local y entonces siento que eso también se veía reflejado en el lenguaje.

[I also feel that, epistemologically—because we were working on the topic of traditional medicines—they [the Canadian group members) understood the topic from a Western science, Global North perspective, which does not hold the same meanings as an Indigenous perspective, or a local perspective, and I feel that this was reflected in language also.

## Conclusion

Findings highlight how despite technological challenges associated with online group work and machine translation software, participants valued cross-cultural and multilingual collaboration. Further analysis is needed to better understand the promises and pitfalls for machine translation software to facilitate multilingual group work in postsecondary education. While recruitment sought to achieve equal representation of English-speaking and Spanish-speaking students, this was not possible due to linguistic imbalances in the population participating in the pilot. Moreover, detailed demographic data was not available for participants, so it was not possible to explore potential influences of gender, age, or other demographics on shaping experiences with machine translation. Through this pilot project and qualitative evaluation, we explored: (1) how machine translation can be harnessed to improve online, international global health education, and (2) to explore the experiences of students utilizing machine translation software to conduct multilingual group work. While there is extensive technical literature describing the potential uses of machine translation, limited research explores the implementation of speech recognition technology in postsecondary education. This research addresses this gap, and underscores the potential for machine translation powered by artificial intelligence to circumvent inequities resulting from the use of English as the de facto language of instruction in global and public health education.

## Data Availability

Not applicable.
